# Lidocaine Spray Versus Viscous Lidocaine Solution Plus Lidocaine Spray in Patients Undergoing Non‐Sedated Esophagogastroduodenoscopy: A Randomized Controlled Trial

**DOI:** 10.1002/deo2.70393

**Published:** 2026-08-05

**Authors:** Natee Faknak, Anuch Singhattha, Thidarat Inpen, Papatsakorn Nopjaroonsri

**Affiliations:** ^1^ Division of Medicine Sawanpracharak Hospital Nakhon Sawan Thailand; ^2^ Gastroenterology Unit, Division of Medicine Sawanpracharak Hospital Nakhon Sawan Thailand

**Keywords:** anesthesia local, esophagogastroduodenoscopy, lidocaine, pharyngeal anesthesia, topical anesthesia

## Abstract

**Background:**

Non‐sedated esophagogastroduodenoscopy (EGD) is preferred in patients at risk from sedatives. However, achieving effective pharyngeal anesthesia remains a challenge. This study compared lidocaine spray alone with a combination of lidocaine spray and viscous lidocaine solution.

**Methods:**

This single‐center, randomized controlled trial was conducted between January 2024 and October 2024 at Sawanpracharak Hospital, Thailand. A total of 250 patients undergoing non‐sedated EGD were randomized to receive lidocaine spray alone (S group, *n* = 125) or lidocaine spray plus viscous lidocaine (S + V group, *n* = 125). The primary outcome was procedural pain measured using a visual analog scale (VAS, 0–10) immediately after completion of the procedure. Secondary outcomes included patient satisfaction, endoscopist satisfaction, ease of intubation, tolerance, and adverse events.

**Results:**

Baseline characteristics were comparable between groups. The S + V group reported significantly lower procedural pain than the S group (mean VAS 1.6 ± 1.1 vs. 3.1 ± 1.7; *p* = 0.01). Patient satisfaction was higher in the S + V group (very satisfied: 23.2% vs. 12.8%; *p* = 0.016). Ease of intubation was also improved (effortless intubation: 43.2% vs. 29.6%; *p* = 0.030). Endoscopist satisfaction numerically favored the S + V group but did not reach statistical significance (*p* = 0.070). No serious adverse events were observed.

**Conclusion:**

Combining lidocaine spray with viscous lidocaine solution significantly reduced procedural discomfort and improved procedural conditions during non‐sedated EGD compared with lidocaine spray alone.

**Trial Registration:**

ClinicalTrials.gov identifier: NCT06185933

## 1 Introduction

Non‐sedated esophagogastroduodenoscopy (EGD) is widely performed in Thailand and many countries due to cost considerations, limited anesthesia resources, patient risk, and patient preference [1, 2, 3]. However, patient discomfort during oropharyngeal intubation remains a major limitation [4, 5]. Topical pharyngeal anesthesia plays a crucial role in improving tolerance to non‐sedated EGD [6, 7]. Lidocaine spray is commonly preferred because of its rapid onset of action and ease of targeted application to the posterior pharynx; however, its relatively short mucosal contact time may limit the duration of anesthetic effect. In contrast, viscous lidocaine solution provides prolonged mucosal exposure and potentially broader anesthetic coverage, but its use may be associated with less precise distribution [8, 9]. Previous studies comparing lidocaine spray and viscous lidocaine solution have generally reported favorable outcomes with lidocaine spray regarding patient tolerance and procedural performance [1, 2]. However, data comparing lidocaine spray alone with the combination of lidocaine spray and viscous lidocaine solution remain limited. A randomized trial by Hayashi et al. found no significant benefit of combination therapy during transoral endoscopy [7]. Given the complementary pharmacologic characteristics of lidocaine spray and viscous lidocaine solution, combining these two formulations may enhance pharyngeal anesthesia by providing both rapid onset and sustained mucosal coverage. However, data evaluating the clinical efficacy of this combination in non‐sedated EGD remain limited [10, 11, 12, 13]. To date, limited data are available directly comparing lidocaine spray alone with the combination of lidocaine spray and viscous lidocaine solution; therefore, this study aimed to evaluate the efficacy of these two techniques in patients undergoing non‐sedated EGD.

## 2 Methods

### 2.1 Study Design and Participants

This study was designed as a parallel‐group, assessor‐blind, randomized controlled trial conducted at Sawanpracharak Hospital, Thailand, between January 2024 and December 2024. Participants were randomly assigned to receive either lidocaine spray alone (S group) or a combination of lidocaine spray and viscous lidocaine solution (S + V group). Participants were randomized in a 1:1 ratio using computer‐generated allocation sequences concealed in sealed opaque envelopes. Allocation occurred in the pre‐procedure area, which was physically separated from both the endoscopy room and the recovery area. Both the endoscopists and the research assistant involved in data collection were blinded to group allocation.

Pharyngeal anesthesia was administered in the pre‐procedure room by trained nurses, without the presence of the endoscopist or research assistant. A blinded research assistant was present during the procedure and in the recovery area to record procedural parameters and collect post‐procedure questionnaire data. All endoscopic examinations were performed using a standard video esophagogastroduodenoscope (EG‐760CT; Fujifilm, Tokyo, Japan). Patients were continuously monitored according to standard practice, including noninvasive blood pressure measurement, heart rate and rhythm via single‐lead electrocardiography, and peripheral oxygen saturation by pulse oximetry. No additional premedications were administered prior to the procedure.

Eligible participants included adults aged 18–80 years undergoing scheduled EGD without sedation. Exclusion criteria included a request for intravenous sedation, any clinical evidence of hepatic encephalopathy, American Society of Anesthesiologists (ASA) physical status of Class IV or V, noncooperation, and refusal to participate in the study.

### 2.2 Pharyngeal Anesthesia Technique

Group S (lidocaine spray alone): Patients received topical anesthesia using 10% lidocaine spray (Oxacain 10% Spray; Sriprasit Pharma, Samut Sakhon, Thailand). A total of five sprays were applied to the oropharynx, with each spray delivering 10 mg of lidocaine. Application was performed using a laryngoscope or tongue depressor to facilitate adequate visualization of the posterior pharynx.

Group S + V (lidocaine spray plus viscous lidocaine): Patients initially received 10% lidocaine spray (five sprays; total dose 50 mg) administered in the same manner as in Group S. This was followed by gargling 5 mL of 2% viscous lidocaine solution (20 mg/mL; lidocaine hydrochloride 2%, hospital‐prepared formulation, Sawanpracharak Hospital, Nakhon Sawan, Thailand). Patients were instructed to retain the solution in the oropharynx without swallowing for at least 1 min to ensure adequate mucosal exposure, after which swallowing was permitted. In both groups, the cumulative lidocaine dose was limited to a maximum of 5 mg/kg. Five minutes after application of lidocaine, patients were transferred to the endoscopy room to undergo the procedure in both groups.

### 2.3 Procedural and Post‐Procedural Assessment

The endoscopist performing the examination was blinded to the assigned pharyngeal anesthesia regimen. During the procedure, the blinded endoscopist, who had been trained in the predefined assessment criteria before study initiation, evaluated the ease of endoscope insertion using a four‐point ordinal scale: 1 = effortless, 2 = easy, 3 = fair, and 4 = difficult. Immediately after completion of the procedure, the assistant completed a standardized questionnaire assessing patient pain using a visual analog scale (VAS) ranging from 0 (no pain) to 10 (worst imaginable pain), tolerance, and overall satisfaction. Patient tolerance was graded as follows: 1 = exceptional, 2 = good, 3 = fair, and 4 = poor. Endoscopist and patient satisfaction with the anesthetic technique were rated on a four‐point scale: 1 = very satisfied, 2 = satisfied, 3 = neutral, and 4 = dissatisfied. Throughout the procedure and the immediate post‐procedural period, patients’ vital signs—including blood pressure, heart rate, cardiac rhythm, and oxygen saturation—were continuously monitored and recorded by blinded nursing staff. The assessment scales for ease of intubation, patient tolerance, and satisfaction were adapted from previously published studies evaluating topical pharyngeal anesthesia for upper gastrointestinal endoscopy [1].

## 3 Outcome Measures

The primary outcome measure was procedural pain intensity, assessed using a VAS ranging from 0 (no pain) to 10 (worst imaginable pain).

Secondary outcome measures included patient satisfaction, endoscopist satisfaction, ease of endoscope insertion, patient tolerance during the procedure, and the incidence of adverse events, including hemodynamic changes (alterations in heart rate and blood pressure), oxygen desaturation (SpO_2_ < 90%), and procedure‐related symptoms (nausea, vomiting, and palpitations).

### 3.1 Statistical Analysis

Sample size was calculated on the basis of a pilot study targeting a mean VAS pain score difference of 1.5 points (SD = 2.0) between groups, requiring 125 patients per group for 80% power at a two‐sided *α* of 0.05. A total of 250 participants (125 per group) were enrolled. Continuous variables were summarized as mean ± standard deviation or median (interquartile range), as appropriate. Categorical variables were summarized as frequency and percentage. Between‐group comparisons were performed using the independent *t*‐test, chi‐square test, or Fisher's exact test, as appropriate. Ordinal outcomes were additionally assessed using trend analysis. Statistical analyses were performed using SPSS version 26.0 (IBM Corp., Armonk, NY, USA). A two‐sided *p* value of <0.05 was considered statistically significant.

## 4 Results

### 4.1 Baseline Characteristics

**FIGURE 1:**
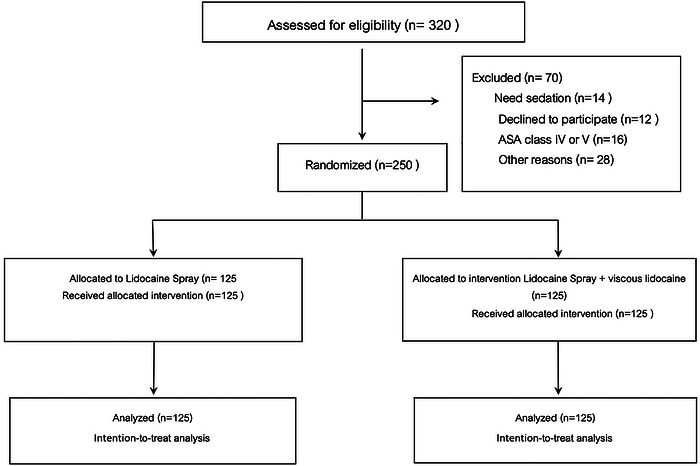
CONSORT participant flow diagram of randomized controlled trial. ASA, American Society of Anesthesiologists.

A total of 320 patients were screened for eligibility, of whom 70 were excluded before randomization. The remaining 250 patients were randomized equally to the lidocaine spray alone group (*n* = 125) or the lidocaine spray plus viscous lidocaine group (*n* = 125). All randomized participants received the allocated intervention and were included in the final intention‐to‐treat analysis (Figure 1). All procedures were performed as outpatient diagnostic EGDs, and no therapeutic endoscopic interventions were performed. The mean age was 61.9 ± 12.5 years in Group S and 58.8 ± 14.6 years in Group S + V, with no statistically significant difference (*p *= 0.076). Procedure duration was also similar between groups (309.1 ± 105.2 s in Group S vs. 297.8 ± 91.4 s in Group S + V; *p *= 0.364). Other baseline clinical variables, including sex distribution, body weight, ASA physical status, and indications for EGD, were well balanced. Indications for EGD were comparable between groups, with upper gastrointestinal bleeding and dyspepsia being the most common indications. The proportion of patients undergoing biopsy during EGD was comparable between the two groups (20.8% vs. 22.4%; *p* = 0.677) (Table 1).

**TABLE 1:** Baseline characteristics of patients.

Characteristics	Group S + V (*n* = 125)	Group S (*n* = 125)	*p* value
Age (years), mean ± SD	58.8 ± 14.6	61.9 ± 12.5	0.076
Body weight (kg), mean ± SD	60.1 ± 12.6	57.7 ± 8.6	0.176
Male gender, *n* (%)	61 (48.8)	54 (43.2)	0.446
ASA physical status, *n* (%)			0.842
ASA I	88 (70.4)	84 (67.2)	
ASA II	30 (24.0)	34 (27.2)	
ASA III	7 (5.6)	7 (5.6)	
Indication for EGD, *n* (%)			0.99
Upper GI bleeding	45 (36.0)	46 (36.8)	
Anemia	24 (19.2)	24 (19.2)	
Dyspepsia	44 (35.2)	45 (36.0)	
Work up malignancy	12 (9.6)	10 (8.0)	
Biopsy performed, *n* (%)	26 (20.8)	28(22.4)	0.677
Procedure time (s), mean ± SD	297.8 ± 91.4	309.1 ± 105.2	0.364

Abbreviations: ASA, American Society of Anesthesiologists; EGD, esophagogastroduodenoscopy.

### 4.2 Procedural Tolerance and Pain

The S + V group reported significantly lower procedural pain (VAS: 1.6 ± 1.1 vs. 3.1 ± 1.7; *p* = 0.01). Patients in Group S + V demonstrated significantly better procedural tolerance compared with Group S (*p* < 0.001). A higher proportion of patients in Group S + V reported very good tolerance (41.6% vs. 25.6%) and good tolerance (50.4% vs. 41.6%), whereas fewer patients reported fair (8.0% vs. 25.6%) or poor tolerance (0.0% vs. 7.2%) (Table 2).

**TABLE 2:** Patient tolerance, satisfaction, and adverse events.

Outcome	Group S + V (*n* = 125)	Group S (*n* = 125)	*p* value
Procedural pain (VAS), mean ± SD	1.6 ± 1.1	3.1 ± 1.7	0.01
Patient satisfaction, *n* (%)			0.016
Very satisfied	29 (23.2)	16 (12.8)	
Satisfied	87 (69.6)	93 (74.4)	
Neutral	9 (7.2)	16 (12.8)	
Dissatisfied	0 (0.0)	0 (0.0)	
Patient tolerance, *n* (%)			<0.001
Very good	52 (41.6)	32 (25.6)	
Good	63 (50.4)	52 (41.6)	
Fair	10 (8.0)	32 (25.6)	
Poor	0 (0.0)	9 (7.2)	
Sore throat, *n* (%)	14 (11.2)	25 (20.0)	<0.001
Nausea/vomiting, *n* (%)	7 (5.6)	9 (7.2)	0.586
Palpitation, *n* (%)	6 (4.8)	4 (3.2)	0.603

Abbreviation: VAS, visual analog scale.

### 4.3 Patient Satisfaction and Endoscopist Assessment

Overall patient satisfaction was high in both groups (*p* = 0.016). A greater proportion of patients in Group S + V reported being very satisfied (23.2% vs. 12.8%), whereas fewer reported neutral satisfaction (7.2% vs. 12.8%). No patients in either group reported dissatisfaction. Endoscopist assessment favored the combination group. Group S + V demonstrated improved ease of intubation (*p* = 0.030), with a higher proportion of procedures rated as effortless (43.2% vs. 29.6%) and fewer rated as fair (4.8% vs. 29.6%) or difficult (0.0% vs. 1.6%). Endoscopist satisfaction was higher in Group S + V (*p* = 0.070), with more procedures rated as very satisfactory (47.2% vs. 35.2%) and fewer rated as neutral (4.8% vs. 25.6%), although this difference did not reach statistical significance (Table 3).

**TABLE 3:** Endoscopist outcome.

Outcome	Group S + V (*n* = 125)	Group S (*n* = 125)	*p* value
Ease of intubation, *n* (%)			0.030
Effortless	54 (43.2)	37 (29.6)	
Easy	65 (52.0)	49 (39.2)	
Fair	6 (4.8)	37 (29.6)	
Difficult	0 (0.0)	2 (1.6)	
Endoscopist satisfaction, *n* (%)			0.070
Very satisfied	59 (47.2)	44 (35.2)	
Satisfied	60 (48.0)	47 (37.6)	
Neutral	6 (4.8)	32 (25.6)	
Dissatisfied	0 (0.0)	2 (1.6)	

### 4.4 Safety and Adverse Events

Hemodynamic parameters remained stable in both groups, with no statistically significant differences observed. Minor adverse events were comparable between groups. Sore throat was significantly less frequent in Group S + V (11.2% vs. 20.0%; *p* < 0.001), whereas nausea/vomiting (69.6% vs. 63.2%, *p* = 0.286) and palpitations (4.8% vs. 3.2%; *p* = 0.603) did not differ significantly. No serious adverse events were observed.

## 5 Discussion

This randomized controlled trial demonstrated that combining lidocaine spray with viscous lidocaine solution significantly improved patient comfort during non‐sedated EGD compared with lidocaine spray alone. Patients in the combination group reported lower procedural pain scores on the VAS (1.6 ± 1.1 vs. 3.1 ± 1.7; *p* = 0.01), representing a clinically meaningful reduction in discomfort. In addition, combination therapy was associated with better procedural tolerance, higher patient satisfaction, and improved ease of endoscope intubation. Together, these findings suggest that enhanced topical pharyngeal anesthesia can substantially improve the overall experience of non‐sedated EGD.

Our findings are consistent with and extend previous studies evaluating topical anesthesia for upper gastrointestinal endoscopy. Amornyotin et al. reported that lidocaine spray alone was superior to viscous lidocaine solution in terms of procedural completion, ease of intubation, and patient satisfaction [1]. Similarly, a meta‐analysis by Watanabe et al. concluded that lidocaine spray was probably more effective than viscous lidocaine alone, while highlighting heterogeneity among studies and the need for further investigation of optimal anesthetic strategies [2]. By combining both formulations, our study suggests that additional benefits may be achieved beyond either modality alone.

Not all prior studies have shown superiority of combination therapy. Hayashi et al. found no significant difference between lidocaine spray alone and combination therapy in a Japanese randomized trial, with comparable pharyngeal observation success rates (100% vs. 100%), procedure times (67 vs. 64 s), and patient discomfort scores (median VAS: 1.0 vs. 1.0) [7]. However, that study focused primarily on pharyngeal observation rather than complete diagnostic EGD, which may require less intense or shorter duration anesthesia. In contrast, our study evaluated patient discomfort during the entire EGD procedure, which involves more prolonged and repeated stimulation of the oropharynx and upper gastrointestinal tract. Therefore, the prolonged mucosal contact provided by viscous lidocaine may have contributed additional anesthetic benefit beyond that achieved with lidocaine spray alone. Furthermore, our primary endpoint was procedural pain assessed by VAS, whereas Hayashi et al. primarily evaluated procedural feasibility and pharyngeal observation outcomes. These differences in procedural endpoints and outcome measures may partially explain the contrasting findings.

The beneficial effects observed in our study are biologically plausible. Lidocaine spray provides rapid onset of anesthesia and targeted delivery to the posterior pharynx, facilitating early suppression of the gag reflex. In contrast, viscous lidocaine solution offers broader mucosal coverage and prolonged contact time [14]. Combining these formulations may therefore provide both immediate and sustained anesthetic effects, resulting in improved comfort and easier endoscope passage.

These findings are particularly relevant in regions where non‐sedated EGD remains common because of cost considerations, limited anesthesia resources, shorter recovery time, and patient preference [13, 15, 16]. In such settings, a simple and inexpensive intervention that improves tolerance may increase patient acceptance of unsedated endoscopy and enhance workflow efficiency in high‐volume endoscopy units.

Importantly, the safety profile was comparable between groups. Hemodynamic parameters remained stable, and no serious adverse events or signs of lidocaine toxicity were observed (Table 4). Minor adverse events were generally similar, although sore throat occurred less frequently in the combination group. These findings support the clinical feasibility of the combined approach when standard dosing precautions are followed.

**TABLE 4:** Changes in hemodynamic parameters.

Parameter	Group S + V (*n* = 125)	Group S (*n* = 125)	*p* value
Systolic BP change, mmHg, median (range)	−0.5 (−14 to 14)	8 (−18 to 43)	0.234
Diastolic BP change, mmHg, median (range)	7.5 (−11 to 26)	1 (−26 to 13)	0.133
Heart rate change, bpm, median (range)	−2 (−20 to 8)	2 (−12 to 26)	0.091
Respiratory rate change, median (range)	0 (0–2)	0 (0–2)	0.610
Oxygen saturation change, %, median (range)	0 (0–2)	0 (0–2)	0.420

This study has several strengths, including its randomized controlled design, adequate sample size, blinded outcome assessment, and evaluation of both patient‐reported and procedural outcomes. Nevertheless, some limitations should be acknowledged. First, this was a single‐center study, which may limit generalizability to other populations and practice settings. Second, all procedures were performed by experienced endoscopists, and results may differ among less experienced operators. Third, the total lidocaine dose differed between the two groups, with patients in the combination group receiving a higher cumulative dose. Therefore, the observed benefits may have been influenced not only by the combined formulations but also by the greater total lidocaine dose. Furthermore, participants were not blinded to treatment allocation, and all outcomes were primarily subjective. Although the endoscopists were intended to remain blinded, residual viscous lidocaine in the oropharynx may have inadvertently revealed treatment allocation during scope insertion, potentially influencing subjective endoscopist‐reported outcomes such as ease of intubation and endoscopist satisfaction. Therefore, expectation bias, previous EGD experience, and unmeasured objective indicators, such as gag reflex frequency or retching episodes, may have influenced the observed outcomes. Further multicenter studies using equivalent total lidocaine doses may help confirm these findings and better define the optimal topical anesthesia regimen for routine clinical practice.

## 6 Conclusion

Combining viscous lidocaine with lidocaine spray improves procedural tolerance, patient satisfaction, and ease of intubation during non‐sedated EGD compared to lidocaine spray alone, without increasing adverse events. This approach represents a simple and practical strategy to enhance patient experience, particularly in settings where non‐sedated EGD is commonly performed Supporting Information.

## Author Contributions

All authors contributed to study conception, data collection, drafting, and approval of the final manuscript. Interpreting data: Natee Faknak. Drafting the manuscript: Natee Faknak. Critical revision of the manuscript: Natee Faknak and Papatsakorn Nopjaroonsri. Statistical analysis: Natee Faknak. Study supervision: Natee Faknak and Papatsakorn Nopjaroonsri.

## Funding

This study was supported by the Medical Education Center, Sawanpracharak Hospital, Nakhon Sawan, Thailand.

## Ethics Statement

Approval of the research protocol: The study protocol was approved by the Institutional Review Board of Sawanpracharak Hospital, Nakhon Sawan, Thailand (approval no. COA 11/2023).

## Consent

Written informed consent was obtained from all participants.

## Conflicts of Interest

The authors declare no conflicts of interest.

## Supporting information




**Supporting File 1**: Data S1 CONSORT checklist.

## Data Availability

The data that support the findings of this study are available from the corresponding author, N.F., upon reasonable request.
